# Reframing Recovery: Fantasy Sports Metrics as Scalable Tools for Measuring Post-injury Athletic Performance

**DOI:** 10.7759/cureus.91602

**Published:** 2025-09-04

**Authors:** William J Gadbois, Hunter Cohn, Rahul Vaidya

**Affiliations:** 1 Medical Education, Wayne State University School of Medicine, Detroit, USA; 2 Orthopedic Surgery, Wayne State University Detroit Medical Center, Detroit, USA

**Keywords:** fantasy sports, injury recovery, post-injury performance, professional sports injuries, return to pre-injury performance

## Abstract

While return to play has traditionally been the benchmark for evaluating athlete recovery after injury, it does not adequately capture whether athletes regain their prior level of performance. The concept of return to pre-injury performance (RPIP) offers a more meaningful measure but lacks a standardized approach for quantification across sports. This narrative review examined the literature using fantasy sports scores and wins above replacement (WAR) to evaluate post-injury performance among professional athletes and to assess their utility as foundations for RPIP. A targeted PubMed search identified studies that used fantasy scores or WAR to quantify post-injury performance in the National Football League (NFL), National Basketball Association (NBA), National Hockey League (NHL), and Major League Basketball (MLB), requiring stratification by injury and use of publicly available scoring systems. Six studies encompassing 15 sport-specific cohorts and 785 athletes met the inclusion criteria. Fantasy metrics and WAR consistently reflected post-injury performance declines in 14 of 15 cohorts across different sports and injury types, despite variation in study design, scoring systems, and follow-up windows. However, only two studies explicitly referenced RPIP. Fantasy scores and WAR may serve as scalable tools for quantifying post-injury performance and provide a preliminary framework for standardizing RPIP across sports. Their public availability, longitudinal structure, and comprehensiveness make them uniquely suited for research, clinical, and operational use. Formalizing RPIP around these metrics could significantly enhance recovery evaluation and decision-making in sports medicine.

## Introduction and background

Injuries are common in professional sports yet determining when and how an athlete should return to sport after an injury remains a complex challenge. Significant research in sports medicine and performance analytics has focused on return to play (RTP) - the point at which an athlete resumes competition [1-5]. While RTP is a convenient and objective milestone, it is a blunt instrument when it comes to evaluating true recovery. The ability to step back onto the field or court does not necessarily imply that the athlete has regained their previous level of function, impact, or value. This distinction underpins the need for more precise metrics that capture recovery beyond mere participation.

Many studies have attempted to assess post-injury performance, often comparing athletes’ pre- and post-injury outputs using individual statistics like points, yards, or minutes played [6-10]. However, these approaches vary widely in methodology and metric selection, making it difficult to compare results across studies, sports, or player positions. To address this, we propose a formalized term - return to pre-injury performance (RPIP) - to describe the percentage of a player’s prior output that is regained after injury. The concept itself is not new, but by framing it under a consistent label, RPIP can serve as a more unified benchmark for assessing recovery quality. For instance, a wide receiver who returns at 80% of his previous output is in a different recovery category than one who returns at 50%, even if both restart playing after comparable recovery periods. 

Fantasy sports scoring systems - and their analytical counterparts like wins above replacement (WAR) - provide a strong foundation for RPIP. These composite scores aggregate a range of performance metrics into a single, interpretable value that is consistent across time and roles. Unlike isolated statistics, fantasy scores allow for a holistic view of performance while remaining publicly available, longitudinally trackable, and sport-specific. By applying RPIP to these scoring systems, we unlock a scalable and objective approach to measuring post-injury performance that can transcend the limitations of current individualized analyses. 

In recent years, several studies have begun to explore this idea [11-16]. Investigations spanning football, basketball, baseball, and hockey have used fantasy scores or WAR to evaluate player performance after injuries, including anterior cruciate ligament (ACL) tears, Tommy John surgery, spinal conditions, and tibial fractures. These studies suggest that fantasy metrics may reflect meaningful post-injury changes, but to date, their application has been siloed by sport, injury type, or scoring method. No review has yet synthesized these findings to evaluate the broader validity and generalizability of fantasy scores as a standardized return-to-performance metric.

In this review, we examine prior research where fantasy scores have been used to evaluate return from injury across the four major North American professional leagues: the National Football League (NFL), National Basketball Association (NBA), National Hockey League (NHL), and Major League Baseball (MLB). We catalog the methodologies of published studies, assess whether fantasy metrics have reliably captured post-injury declines, and explore whether they have been framed as tools for assessing RPIP rather than just participation. We hypothesize that fantasy scores, due to their structure and relevance, are a valid and scalable surrogate for evaluating true return to performance across sports and injury types.

## Review

Methods

This review was designed to evaluate how fantasy sports scores and WAR have been used to measure post-injury performance, particularly as proxies for RPIP, across major North American professional sports.

Search Strategy and Study Identification

We conducted a targeted literature review to identify studies evaluating the use of fantasy sports scores or WAR as metrics of athlete performance following injury. Searches were performed in PubMed using Boolean logic that combined terms related to fantasy sports (e.g., “fantasy football,” “fantasy points,” “wins above replacement”) with terms related to athletic performance, recovery, and return to pre-injury performance (e.g., “return to play,” “post-injury performance,” “player performance”).

The final Boolean search string included the following:

("fantasy sports"[All Fields] OR "fantasy points"[All Fields] OR "wins above replacement"[All Fields] OR "fantasy football"[All Fields] OR "fantasy baseball"[All Fields] OR "fantasy basketball"[All Fields] OR "fantasy hockey"[All Fields]) AND Points= (1*Points) + (1*Three Pointers Made) − (1×Field Goals Attempted) + (2×Field Goals Made) − (1×Free Throws Attempted) + (1×Free Throws Made) + (1×Rebounds) + (2×Assists) + (4×Steals) + (4×Blocks) − (2×Turnovers) ("return to play"[All Fields] OR "return-to-play"[All Fields] OR "postinjury performance"[All Fields] OR "player performance"[All Fields] OR "rehabilitation"[All Fields] OR "performance metrics"[All Fields]) 

Additional studies were identified through manual review of references and forward citation tracking. We limited our search to PubMed-indexed, peer-reviewed literature. Gray literature (e.g., blogs, sports analytics sites) was intentionally excluded because our objective was to synthesize evidence from studies that had undergone formal peer review, ensuring greater methodological rigor and reproducibility. Moreover, a cursory review of gray literature sources did not identify substantial publications relevant to the topic. 

**Figure 1:**
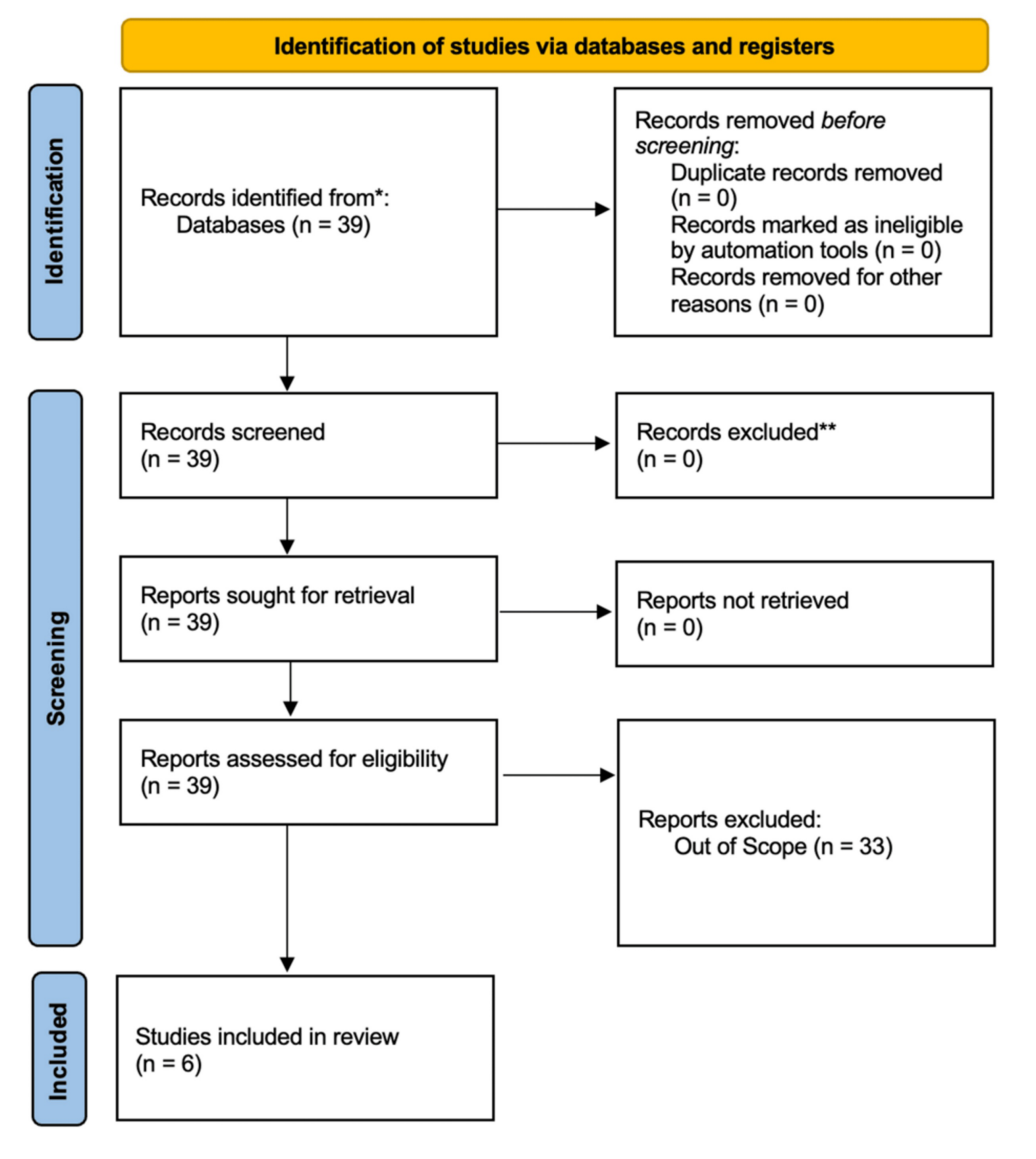
PRISMA flow diagram of study selection PRISMA: Preferred Reporting Items for Reviews and Meta-Analyses.

This study was conducted as a narrative review rather than a systematic review. A narrative design was selected because the body of literature is small, heterogeneous in design, and exploratory in nature, making meta-analysis or systematic synthesis impractical. Nonetheless, Preferred Reporting Items for Systematic Reviews and Meta-Analyses (PRISMA) guidelines for narrative reviews were followed where applicable, including transparent reporting of search strategy, inclusion criteria, and study selection. A PRISMA-style flow diagram (Figure 1) is included to enhance transparency.

Inclusion and Exclusion Criteria 

To be eligible for inclusion, studies were required to focus on professional athletes from one of the four major North American sports leagues: the NFL, NBA, MLB, or NHL. Additionally, studies had to examine athlete performance following a clearly defined injury or surgical intervention. A central criterion was the use of fantasy points or WAR as a primary or secondary metric to quantify post-injury performance. Only studies that relied on publicly available data sources for both fantasy scoring and performance metrics were considered. Conversely, studies were excluded if they did not stratify outcomes by injury type, used fantasy metrics solely for predictive modeling purposes such as betting or fantasy projections, or if their populations were limited to amateur or collegiate athletes. 

Overview of fantasy scoring systems

Fantasy sports scoring systems are built upon real-world performance statistics, with each athlete’s game output translated into a point total based on predefined rules. While scoring formulas vary slightly across platforms (e.g., ESPN, Yahoo, FantasyData), they consistently aggregate contributions into a single composite score that reflects productivity, efficiency, and negative plays.

Fantasy scores can be conceptually divided into three categories as depicted in Table 1.

**Table:** 

Scoring Dimension	Description
Productivity	These reflect high-impact offensive contributions such as touchdowns, goals, home runs, runs batted in, or assists.
Efficiency	These account for performance per opportunity - such as yards per carry, field goal percentage, innings pitched, or shooting accuracy.
Negative Events	These deduct points for detrimental plays, including interceptions, fumbles, turnovers, penalty minutes, or earned runs allowed.

Table 1: Fantasy Scoring Dimensions and Descriptions

This table outlines the three primary dimensions used in fantasy sports scoring systems: productivity, efficiency, and negative events.

Each major sport utilizes these components differently. Examples of statistics considered are presented in Table 2, followed by the equations used to calculate fantasy scoring for each sport.

**Table:** 

Sport	Scoring System	Statistics Considered
NFL	Standard fantasy point system	Passing, rushing, and receiving touchdowns; yardage (25 yards passing, 10 yards rushing/receiving); two-point conversions; deductions for fumbles and interceptions
NBA	Nine-category format	Points, rebounds, assists, steals, blocks, turnovers, field goal percentage (FG%), free throw percentage (FT%), three-point percentage (3P%)
MLB	Traditional fantasy scoring or WAR	Hits, runs, strikeouts, earned runs, walks (fantasy); batting, baserunning, fielding, positional value, and league adjustment (WAR)
NHL	Position-adjusted scoring	Goals, assists, penalty minutes, plus-minus, power play contributions, saves, and shutouts

Table 2: Sport-Specific Fantasy Scoring Systems and Metrics

This table summarizes how each major professional sport (NFL, NBA, MLB, NHL) applies fantasy scoring systems, highlighting both the type of scoring format used and the specific statistics considered.

NFL: National Football League, NBA: National Basketball Association, NHL: National Hockey League, and MLB: Major League Baseball.

*General Fantasy Scoring System by Sport* 

NFL:

$\begin{aligned} \text{Offensive Points} =\;& (6 \times \text{Touchdowns}) + \left(\frac{\text{Pass Yards}}{20}\right) \\ &+ \left(\frac{\text{Rush Yards}}{10}\right) + \left(\frac{\text{Receiving Yards}}{10}\right) \\ &- (2 \times \text{Interceptions}) - (1 \times \text{Fumbles}) \\ \end{aligned}$

$\begin{aligned}\text{Defensive Points} =\;& 2 \times (\text{Safety} + \text{Sack} + \text{Interceptions} + \text{Fumble Recoveries}) \\ &+ 6 \times \text{Defensive Touchdowns}\\\end{aligned}$

NHL:

$\begin{aligned}\text{Forward Points} =\;& \left(\frac{\text{Penalty Minutes}}{4}\right) + (4 \times \text{Goals}) + (3 \times \text{Assists}) \\ &+ (2 \times \text{Power play Goals}) + (3 \times \text{Game Winning Goals}) \\ &+ (1 \times \text{Plus/Minus}) + (3 \times \text{Shorthanded Goals}) \\ \end{aligned}$

$\begin{aligned}\text{Defenseman Points} =\;& \left(\frac{\text{Penalty Minutes}}{4}\right) + (5 \times \text{Goals}) \\ &+ (4 \times \text{Assists}) + (2 \times \text{Power play Goals}) + (3 \times \text{Game Winning Goals}) \\ &+ (1 \times \text{Plus/Minus}) + (3 \times \text{Shorthanded Goals}) \\ \end{aligned}$

$\begin{aligned}\text{Goalie Points} =\;& (2 \times \text{Wins}) + (1 \times \text{Overtime Losses}) + (1 \times \text{Goals}) \\ &+ (1 \times \text{Assists}) + (2 \times \text{Shutouts}) + \left( \frac{\text{Saves}}{5} \right) \\ &+ (1 \times \text{Losses}) - \left(0.75 \times \text{Goals Allowed}\right) \\ \end{aligned}$

NBA:

$\begin{aligned}\text{Points} =\;& (1 \times \text{Points}) + (1 \times \text{Three Pointers Made}) \\ &- (1 \times \text{Field Goals Attempted}) + (2 \times \text{Field Goals Made}) \\ &- (1 \times \text{Free Throws Attempted}) + (1 \times \text{Free Throws Made}) \\ &+ (1 \times \text{Rebounds}) + (2 \times \text{Assists}) \\ &+ (4 \times \text{Steals}) + (4 \times \text{Blocks}) \\ &- (2 \times \text{Turnovers}) \end{aligned}$

MLB:

$\text{WAR} = \frac{\text{Batting Runs} + \text{Base Running Runs} + \text{Fielding Runs} + \text{Positional Adjustment} + \text{League Adjustment} + \text{Replacement Runs}}{\text{Runs Per Win}}$

These fantasy scoring systems offer several advantages for research use. First, they provide consistency, as fantasy scores are generated for every professional game and are not susceptible to loss to follow-up. Second, they offer comprehensiveness, capturing both the volume and efficiency of athletic output across multiple performance domains. Third, they ensure accessibility as fantasy scores are publicly available, easily interpretable by both experts and non-experts, and can be tracked longitudinally across seasons. Taken together, these features make fantasy metrics a promising surrogate for assessing return to performance, with the potential to complement or even replace more subjective clinical or observational measures.

Importantly, while this scoring structure provides a conceptual skeleton, there is variation by platform. For example, FantasyData.com includes scoring for defensive players, and some league or user-defined settings may assign bonus points for long touchdowns, shutouts, or milestone achievements. Another example of variation is demonstrated through different fantasy football scoring formats. One popular model is called points-per-reception (PPR) and gives an extra point for each catch a player makes, compared to half-PPR, or non-PPR leagues. These different point systems shift point distribution, particularly for pass-catching positions, introducing further variation between studies. These variations, however, do not detract from the utility of fantasy scores as a metric - they still represent a comprehensive, standardized snapshot of performance across essential categories and remain consistently applicable for longitudinal and comparative analysis.

Data extraction and synthesis

A high-level overview of the information collected from each article follows, and a full breakdown by sport is shown in Table 3. To assess study quality, we applied the Strengthening the Reporting of Observational Studies in Epidemiology (STROBE) checklist to each included study. All studies met baseline methodological quality standards, although sample size and retrospective design constraints were common. For each study, we collected basic information including the title, authors, year of publication, the sport and injury type studied, the sample size, and the player positions included. We recorded the type of fantasy scoring system used (such as standard scoring or WAR) and where the data came from (e.g., ESPN, Rotoworld, FanGraphs, etc.). Any differences from standard scoring systems were also noted. We documented the study design and analysis method (such as pre/post or matched cohort), the inclusion and exclusion criteria, and the range of years included in the analysis. In addition, we tracked the statistical methods used, the reported effect of injury on fantasy performance, the length of post-injury follow-up, and any other findings related to recovery, such as return to play time, career length, or RPIP.

**Table 1:** Comparative Summary of Included Studies Which Used Fantasy Scores to Evaluate Post-Injury Performance NFL: National Football League, NBA: National Basketball Association, MLB: Major League Baseball, NHL: National Hockey League, ACL: anterior cruciate ligament, UCL: ulnar collateral ligament, WAR: wins above replacement, 3P FG: three-point field goal, TD: touchdown, FG: Field goal, PAT: point after touchdown, 2P FG: two-point field goal, FT: free throw, QB: quarterback, RB: running back, WR: wide receiver, TE: tight end, ANOVA: analysis of variance, ANCOVA: analysis of covariance, RTP: return to play, RPIP: return to pre-injury performance

Author and Publication Date	Sport(s) Studied	Injury Type/Condition	Sample Size	Positions Included	Fantasy Stat/WAR Source	Stats included in fantasy scoring system which vary from our methods (e.g. Sacks, Team Win)	Study Design	Analysis Method	Inclusion/Exclusion Criteria	Analyzed Season Year Range for Included Players	Statistical Analysis Methods (e.g. t-tests, Spearman rho, etc.)	Change in Fantasy Performance/WAR Post-Injury	Any other correlations found?	Other Post-Injury Metrics Included	Length of Post-injury Investigation
Kajy et al. 2023 [12]	NFL	ACL Tear	25	Not Specified	Rotoworld.com	Field goals, Extra points, Sacks, Safeties	Retrospective Cohort	Non-control matching	Must have missed at least one game due to surgery; Can't have gone through more than one ACL reconstructive surgery	1992-2015	N/A	50% of players saw a decline in performance	58% of players saw an increase in games played post-injury	Time to Return to Play (RTP): 11 months; Percentage of Players who RTP: 88%; Return to Pre-Injury Performance Level (RPIP): 36%; Career Length Post-Injury: 26 months	Remainder of career
	NBA	ACL Tear	26	Not Specified	Rotoworld.com	3P FGs	Retrospective Cohort	Non-control matching	Must have missed at least one game due to surgery, and can't have gone thru more than one ACL reconstructive surgery	1992-2015	N/A	54% of players saw a decline in performance	46% of players saw an increase in games played post-injury, while 23% saw a decrease in games played	Time to Return to Play (RTP): 13 months; Percentage of Players who RTP: 85%; Return to Pre-Injury Performance Level (RPIP): 42%; Career Length Post-Injury: 64 months	Remainder of career
	MLB	ACL Tear	15	Not Specified	Fangraphs.com	N/A	Retrospective Cohort	Non-control matching	Must have missed at least one game due to surgery, and can't have gone thru more than one ACL reconstructive surgery	1992-2015	N/A	60% of players saw a decline in performance	47% of players saw an increase in games played post-injury	Time to Return to Play (RTP): 9 months; Percentage of Players who RTP: 93%; Return to Pre-Injury Performance Level (RPIP): 33%; Career Length Post-Injury: 42 months	Remainder of career
	NHL	ACL Tear	17	Forward, Defenseman, Goalie	Rotoworld.com	Short-handed goals, Game winning goals, Wins, Overtime losses, Losses, Goals allowed	Retrospective Cohort	Non-control matching	Must have missed at least one game due to surgery, and can't have gone thru more than one ACL reconstructive surgery	1992-2015	N/A	47% of players saw a decline in performance	17% of players saw a decrease in games played post-injury, while 17% of players didn't return to play	Time to Return to Play (RTP): 8 months; Percentage of Players who RTP: 82%; Return to Pre-Injury Performance (RPIP) Level Time: 14 months; Percentage of Players who RPIP: 35%; Career Length Post-Injury: 37 months	Remainder of career
Simcox et al. 2022 [13]	MLB	UCL tear	216	Pitcher	ESPN.com Yahoo.com Cbssports.com	Hit allowed, Inning pitched, Hit batsman, Quality start	Retrospective Cohort	Non-control matching	Must have started a minimum of 45 games or relief pitch in 90 games in 3 seasons prior to injury	1974-2018	Paired 2-sided t-tests, and ANOVA	Pitchers saw a 37.7% (ESPN, p<0.001), 38.6% (Yahoo, p<0.001), and 36.9% (CBS, p<0.001) decrease in fantasy performance	Players pitched fewer games, fewer innings, and declined in all on-field performance statistics	Time to Return to Play (RTP): 16.6 months; Percentage of Players who RTP: 83%; Average Age of Players at Injury: 30 years; Percentage of Players who played for >3 Seasons Post-Injury: 63%	3 seasons
Burgess et al. 2022 [16]	NFL	ACL Tear	72	Quarterback, Running Back, Wide Receiver	STATS.com Pro-Football-Reference.com	Two-point conversions, Kickoff return TD, Punt return TD, Fumble receovered for TD	Retrospective Cohort	Control-matching	All QB, RB, WR, TE who sustained an isolated unilateral ACL injury from 1988 to 2017 (from NFL injury reports, press releases and internet resources) Players who played ≥3 games before their injury and 3 games after their injury Prior ACL/concomitant ligament injuries in ipsilateral knee were excluded, TEs were excluded	1988-2017	Two-sided t tests	RBs and WR showed significant decreases in performance (p<0.05); QBs did not show any significant difference	Believe fantasy scores are a true objective way to measure performance following injury; recognized by population and athletes alike and easily discernable. PROMs are limited. 21-37% of players with ACL injury never appear in another NFL game (cited from other papers); RTP is lower (other paper)	None	3 seasons
Bergstein et al. 2024 [14]	NFL	Ankle Injury (Ankle sprain, high ankle sprains, bone bruises)	303	Quarterback, Running Back, Wide Receiver	Pro-Football-Reference FantasyData.com	Kick or punt return TD, Fumble return for TD, 2 point conversion	Retrospective Cohort	Cohort-matching	Must have experienced either an ankle sprain, high ankle sprain, or bone bruise; and returned to professional play Must not have multiple injuries in that season, zero fantasy points pre-injury, played fewer than 3 games post-injury, or other injury types	2009-2020	Paired t-tests, independent t-tests (demographics), ANCOVA (adjusted for RTP and injury type, Bonferroni post-hoc analysis	RBs/WRs/TEs showed significant decreases in performance (p<0.0001); QBs did not show any significant difference	RTP time and injury type confounded games played but not fantasy point outcomes; no significant difference by injury subtype	None	1 season
Kajy et al. 2022 [11]	NFL	Lumbar Discectomy/Microdiscectomy	14	All	Rotoworld.com ESPN	Field goals, Extra points, Sacks, Safeties	Retrospective Cohort	Non-matching	Must have missed at least one game due to lumbar discectomy; public records only	1993-2015	Descriptive stats only (means, %); no inferential stats reported	71% of players saw a decline in performance	71% of players saw a decrease in games played. Shortest average post-op career length (34.8 months)	Time to Return to Play: 6 months; Percentage of Players who RTP: 64%; Career Length Post-Injury: 34.8 months; Return to Pre-Injury Performance Time: 15 months	Remainder of career
	NBA	Lumbar Discectomy/Microdiscectomy	7	All	Rotoworld.com	3P FGs	Retrospective Cohort	Non-matching	Must have missed at least one game due to lumbar discectomy; public records only	1993-2015	Descriptive stats only (means, %); no inferential stats reported	71% of players saw a decline in performance; 14% of players saw an increase in performance	1 player saw an increase in games played but a decrease in performance, 1 player saw a decrease in games played but an increase in performance, and 4 players saw a decrease in both. Longest average post-op career length (48 months)	Time to Return to Play: 2.8 months; Percentage of Players who RTP: 86%; Career Length Post-Injury: 48 months; Return to Pre-Injury Performance Time: 12 months	Remainder of career
	MLB	Lumbar Discectomy/Microdiscectomy	8	All	Fangraphs.com	N/A	Retrospective Cohort	Non-matching	Must have missed at least one game due to lumbar discectomy; public records only	1993-2015	Descriptive stats only (means, %); no inferential stats reported	100% of players saw a decline in performance	88% of players saw a decrease in games played post-injury	Time to Return to Play: 12 months; Percentage of Players who RTP: 88% Career Length Post-Injury: 44 months; Return to Pre-Injury Performance Time: 24 months	Remainder of career
	NHL	Lumbar Discectomy/Microdiscectomy	9	Forward, Defenseman, Goalie	Rotoworld.com	Shorthanded goals, Game winning goals, Goals allowed, Wins, Losses, Overtime losses	Retrospective Cohort	Non-matching	Must have missed at least one game due to lumbar discectomy; public records only	1993-2015	Descriptive stats only (means, %); no inferential stats reported	66.6% of players saw a decline in performance	4 players saw a decrease in performance and games played, 2 saw a decrease in performance but an increase in games played, 3 saw an increase in both	Time to Return to Play: 4.7 months; Percentage of Players who RTP: 89%; Career Length Post-Injury: 29 months; Return to Pre-Injury Performance Time: 12 months	Remainder of career
Punreddy et al. 2024 [15]	NFL	Tibial Fracture	14	Running Back, Wide Receiver, Defensive End, Safety, Quarterback, Center	Fantasydata.com Statmuse.com	Defensive stats (e.g. interceptions, fumbles, touchdowns, sacks, tackles, etc.), Special Teams stats (e.g. kick/punt return TDs, PATs made, FGs made)	Retrospective Cohort	Non-control matching	N/A	2015-2023	Pearson correlation	Decrease in performance by 29.1%	No correlation between age, weight, or height and post-injury success	Age at injury: 26.96 years; Time to Return to Play (RTP): 350.18 days	1 season
	NBA	Tibial Fracture	3	Center, Small Forward	Fantasydata.com Statmuse.com	3P FGs, 2P FGs, FT made	Retrospective Cohort	Non-control matching	N/A	2015-2023	Pearson correlation	Decrease in performance by 34.5%	No correlation between age, weight, or height and post-injury success	Age at injury: 26.96 years; Time to Return to Play (RTP): 314.67 days	1 season
	MLB	Tibial Fracture	9	Infielder, Outfielder, Pitcher	Fantasydata.com Statmuse.com	N/A	Retrospective Cohort	Non-control matching	N/A	2015-2023	Pearson correlation	Increase in performance by 8.1%	No correlation between age, weight, or height and post-injury success	Age at injury: 26.96 years; Time to Return to Play (RTP): 234.13 days	1 season
	NHL	Tibial Fracture	2	Center, Right Winger	Fantasydata.com Statmuse.com	Shots on goal, Blocks, Short handed goals/assists, Shootout goals, Wins, Goals against	Retrospective Cohort	Non-control matching	N/A	2015-2023	Pearson correlation	Decrease in performance by 14.1%	No correlation between age, weight, or height and post-injury success	Age at injury: 26.96 years; Time to Return to Play (RTP): 106.5 days	1 season

The findings were compiled into a comparative table and synthesized narratively to assess methodological similarities, differences in scoring systems, and consistent outcome trends across sports and injuries.

Results

Our narrative review identified six studies that evaluated fantasy sports scores or WAR as metrics of post-injury performance across the NFL, NBA, MLB, and NHL. These studies collectively examined 15 sport-specific cohorts (five NFL, four MLB, three NBA, three NHL) and included 785 professional athletes with injuries ranging from ACL tears, lumbar discectomies, ulnar collateral ligament tears (UCL), tibial fractures, and ankle sprains. Below, we synthesize, compare, and contrast their methodologies, findings, and limitations.

Fantasy Scores and WAR Consistently Reflect Post-injury Performance Declines

All six studies reported post-injury performance declines in at least one sport cohort, with 14 of 15 sport-specific analyses demonstrating statistically significant or clinically meaningful reductions in fantasy scores or WAR (Table 4). Table 4 summarizes the number of cohorts by sport and the proportion showing performance decline. Table 5 further breaks down these findings by sport and injury type, highlighting sample sizes and specific positional differences in outcomes. Table 6 consolidates the data by injury type across all sports, indicating whether performance declined for each injury-sport combination. 

**Table 2:** Summary of Post-Injury Performance Declines by Sport Total number of cohorts by sport and the proportion exhibiting a decline in performance following injury.

Sport	Number of cohorts	Nuber of cohorts showing decline
NFL	5	5 (100%)
NBA	3	3 (100%)
NHL	3	3 (100%)
MLB	4	3 (75%)

**Table 3:** Sport-Specific Performance Changes by Injury Type Breakdown of performance outcomes by sport and injury type, including sample sizes and positional differences. ACL: anterior cruciate ligament, QB: quarterback, RB: running back, WR: wide receiver.

Injury type	Sample size	Post-injury performance findings
NFL (5/5 cohorts declined)		
ACL Reconstruction	72 players	50% of players saw a decline in performance; RBs/WRs showed a significant decline in performance (p<0.05); QBs did not show any significant difference
Ankle Injuries	303 players	RBs/WRs showed a significant decline in performance (p<0.0001); QBs did not show any significant difference
Lumbar Discectomy	14 players	71% of players saw a decline in performance
Tibial Fractures	14 players	Decrease in performance by 29.1%
NBA (3/3 cohorts declined)		
ACL Reconstruction	26 players	54% of players saw a decline in performance
Lumbar Discectomy	7 players	71% of players saw a decline in performance
Tibial Fractures	3 players	Decrease in performance by 34.5%
MLB (3/4 cohorts declined)		
ACL Reconstruction	15 players	60% of players saw a decline in performance
Tommy John Surgery	216 players	Decrease in performance by an average of 37.7% (p<0.001)
Lumbar Discectomy	8 players	100% of players saw a decline in performance
Tibial Fractures	9 players	Increase in performance by 8.1%
NHL (3/3 cohorts declined)		
ACL Reconstruction	17 players	47% of players saw a decline in performance
Lumbar Discectomy	9 players	67% of players saw a decline in performance
Tibial Fractures	2 players	Decrease in performance by 14.1%

**Table 4:** Overview of Performance Changes by Injury Type Across Sports Summary of performance outcomes by injury type across all sports, indicating decline by injury-sport combination. NFL: National Football League, NBA: National Basketball Association, MLB: Major League Baseball, NHL: National Hockey League.

Injury investigated	Sports	Performance Decline?
ACL Reconstruction	NFL/NBA/MLB/NHL	Yes
Tommy John Surgery	MLB	Yes
Ankle Injuries	NFL	Yes
Lumbar Discectomy	NFL/NBA/MLB/NHL	Yes
Tibial Fractures	NFL/NBA/NHL	Yes
Tibial Fractures	MLB	No

Of the six studies reviewed, only two specifically addressed RPIP outcomes - one focused on ACL injuries (Kajy et al. [11]) and the other on lumbar discectomy (Kajy et al. [11]). In the ACL study, 30%-40% of players across the four leagues returned to their pre-injury performance levels. Notably, MLB players took the longest to do so, with an average recovery time of 21 months. In the lumbar discectomy study, the average time to return to pre-surgical performance was 15 months for NFL players, 12 months for both NBA and NHL players, and 24 months for MLB players. 

Methodological Approaches 

The included studies employed a range of designs and analytic frameworks to assess post-injury performance, with notable variation in performance windows, comparison groups, and statistical strategies. Post-injury performance windows differed across studies: two studies measured performance during the first full season after return (tibial fracture and ankle injury cohorts), two others used a three-season follow-up period (ACL: Burgess et al. [16]; Tommy John surgery: Simcox et al. [13]), and two assessed performance across the remainder of the athlete’s career (ACL: Kajy et al. [11]; lumbar discectomy cohorts: Kajy et al. [11]). Matched cohort designs were used in studies such as the NFL ankle injury analysis, which included a control group of uninjured players matched by position and performance level - allowing for direct comparisons between injured and non-injured athletes across similar timeframes. Pre-/post-designs were also common, evaluating changes in performance before and after injury within the same player. This approach was applied in the MLB Tommy John and lumbar discectomy studies, as well as in both ACL cohorts. Finally, descriptive cohort designs were utilized in studies with smaller sample sizes, such as those examining NHL or NBA players with spinal surgeries. These studies typically reported group-level averages or percent changes in fantasy scores without formal statistical testing. 

Across designs, consistent findings emerged despite variation in sample size, statistical methods, and scoring platforms. Only the NFL ankle and MLB Tommy John studies used covariate adjustment (e.g., for RTP time) or multiple-testing corrections (Bonferroni). The remaining studies either omitted these safeguards or, in descriptive cases, lacked statistical testing entirely. 

Fantasy scoring systems were largely consistent in how performance was captured. NFL studies used ESPN or FantasyData’s standard scoring systems, while MLB studies used FanGraphs’ WAR, which integrates offense, defense, and positional adjustment. NBA and NHL studies relied on composite point systems tailored by position, incorporating key stat categories such as points, assists, blocks, or saves. However, scoring platform differences (e.g., ESPN vs. Yahoo) were not addressed in any study.

Collective limitations

Several limitations emerged across the included studies. First, sample size constraints were common; nearly half of the cohorts (seven out of 15) included fewer than 20 athletes, such as the NHL tibial fracture analysis (n=2) and the NBA lumbar discectomy group (n=7). These small samples increased the risk of Type II errors and made it difficult to detect meaningful differences, particularly when examining position-specific outcomes. 

Second, the use of retrospective designs introduced potential for selection bias. All but one study (the NFL ankle injury cohort) relied on historical data. In several cases, players who retired following injury were not included in return-to-performance calculations, which may have led to an underestimation of post-injury performance declines. This reflects a broader survivorship bias, as excluding non-returning athletes inflates estimates of RPIP. Publication bias is also possible, as null or unfavorable results may be underreported. 

Third, the small number of published studies (n=6, 785 athletes in total) limits the strength of any claims about the validity or scalability of fantasy metrics. Few studies reported inferential statistics, and effect sizes (e.g., Cohen’s d) were not available for most outcomes. This limited the ability to formally quantify the magnitude of performance declines across studies. Where descriptive data were provided (e.g., Kajy et al.), effect size estimation was not possible due to lack of variance reporting, further constraining interpretability. 

Fourth, there was incomplete metric capture, particularly in how fantasy scores reflected different types of contributions. While WAR incorporated defensive performance in MLB, fantasy scoring in other sports often failed to account for non-scoring contributions, such as offensive line play in the NFL or defensive stops in the NBA. These omissions may undervalue players whose roles are not adequately represented by traditional fantasy metrics.

Discussion

The findings of this narrative review present a compelling case for the validity and utility of fantasy sports metrics and WAR as tools for evaluating post-injury performance in professional athletes. Across six studies encompassing four major North American sports leagues and multiple injury types, these metrics demonstrated remarkable consistency in detecting and quantifying performance declines following injury [11-16]. For instance, fantasy analyses reveal diminished performance post-ACL in NFL running backs and wide receivers [12,16], reduced output after ankle injuries in offensive players [14], and substantial declines (57-100%) following lumbar discectomy across leagues [11]. In MLB, WAR has been used to evaluate post-Tommy John surgery recovery trajectories [13], and declines in performance following UCL reconstruction have been documented using both WAR and traditional stats [15]. This convergence of evidence suggests that fantasy scores and WAR are not merely recreational tools, but serious instruments for athletic assessment. Their consistent use of standardized, composite statistics enables comparisons that are difficult to achieve with isolated performance metrics. In doing so, they provide a scalable and accessible foundation for RPIP - offering a quantifiable way to assess not just whether an athlete has returned, but how much of their prior impact they have regained. As such, fantasy scores warrant broader adoption in sports medicine and performance analysis as an objective underpinning of RPIP frameworks. 

Cross-sport and Cross-injury Consistency

The consistency of performance declines observed across different sports (NFL, NBA, NHL, MLB) and injury types (ACL tears, lumbar discectomy, Tommy John surgery, tibial fractures, ankle sprains) is particularly notable. Despite varied physical demands, healing timelines, and positional roles, fantasy scores and WAR declined post-injury in 14 of 15 cohorts [11-16]. This robustness strengthens the argument that these composite metrics capture an essential element of athletic recovery - one that is not entirely reflected by return-to-play status alone.

What makes these findings particularly persuasive is the diversity of contexts in which fantasy metrics proved effective. The studies reviewed spanned different sports with fundamentally different physical demands, ranging from the explosive movements of NFL receivers to the rotational power required of MLB pitchers. They included a variety of injury types, from acute traumatic events like ACL tears to overuse injuries such as UCL damage. Additionally, the studies utilized multiple performance systems, including both traditional fantasy point models and more advanced sabermetric indices like WAR. Finally, the methodological approaches varied widely, incorporating retrospective cohort studies as well as matched-control designs. Despite these differences, 14 of the 15 sport-specific cohorts demonstrated meaningful declines in fantasy output or WAR following injury. This consistent pattern not only affirms that performance typically suffers post-injury but also highlights fantasy scores and WAR as reliable tools to quantify that decline. By consolidating diverse performance inputs into a single value, these metrics offer a standardized, scalable means of measuring return to pre-injury performance in ways that isolated statistics cannot. 

*Why Fantasy Metrics Excel as Performance Indicators* 

Fantasy scoring systems and WAR possess several inherent advantages over traditional performance metrics, making them particularly well-suited for assessing recovery after injury. One key strength is their comprehensiveness. Unlike single-statistic measures such as batting average or completion percentage, fantasy metrics combine multiple dimensions of performance into a unified score, making them ideal for evaluating the conservative benefits of sports medicine interventions on athlete recovery and performance. For example, an NFL running back’s fantasy production reflects not only rushing yards but also receiving ability, scoring efficiency, and ball security. Similarly, WAR captures a baseball player's overall value by incorporating hitting, fielding, baserunning, positional value, and league context. This multifaceted nature allows these metrics to reflect the complex and varied ways injuries affect overall performance. 

A second advantage is longitudinal tracking. Fantasy scores and WAR are calculated for every game of a player’s career, enabling researchers to establish individual pre-injury baselines and monitor recovery trajectories over time. This level of continuity offers a level of breadth and precision that periodic clinical assessments cannot match. 

Fantasy metrics also offer strong contextual relevance, as they align closely with how teams, fans, and decision-makers evaluate player value. A 15% decline in a point guard’s fantasy production following ACL surgery is not just a statistical artifact, but a real reduction in on-court impact that influences coaching strategies and contract decisions. 

Finally, these tools offer objective benchmarking. Because fantasy scores are derived from official, publicly available statistics, they eliminate much of the subjectivity found in clinical observations or coaching opinions. Two players with identical performance outputs will receive identical fantasy scores, independent of external narratives or biases. Taken together, these advantages reinforce fantasy metrics and WAR as uniquely powerful tools for evaluating post-injury performance. 

*Addressing the Outlier: MLB Tibial Fractures* 

The single exception to the overall pattern of post-injury performance decline - improved outcomes in MLB players following tibial fractures - warrants careful consideration rather than dismissal [15]. Several factors may help explain this anomaly. First, sample characteristics likely played a role, as the cohort was small (n=9), increasing susceptibility to random variation; in such cases, a single player's exceptional recovery could disproportionately influence group averages. Second, the mechanism of injury may be relevant. In baseball, tibial fractures often result from acute traumatic events, such as being hit by a pitch, rather than from overuse or degenerative processes. These acute injuries may allow for cleaner healing and more complete recovery compared to chronic soft tissue injuries. Positional factors could also contribute, as some players may have shifted to roles like designated hitter during recovery - positions that place fewer physical demands on the affected limb and may help preserve or even enhance offensive metrics. Lastly, rehabilitation effects may play a role; longer recovery periods could facilitate compensatory strength gains in unaffected areas, potentially boosting overall performance upon return. Rather than undermining the validity of fantasy metrics, this outlier actually underscores their value. The ability of fantasy scores to capture unexpected trends highlights their sensitivity and suggests they may help identify injury types with unique recovery trajectories and warrant further investigation. 

Limitations and Pathways for Improvement

While the evidence supporting fantasy metrics is strong, several limitations across the included studies present important opportunities for refinement and future research. 

One key limitation involves variation in post-injury performance windows. Some studies measured recovery based solely on the first full season following return, while others extended their analysis to include three seasons or even the remainder of the athlete’s career. These discrepancies complicate direct comparisons across studies and may skew findings depending on the nature of the injury, the athlete's role, or the time needed for performance stabilization [17, 18]. Moving forward, standardizing performance windows, or at a minimum, clearly contextualizing them based on injury type and player position, will be essential to improve consistency and enable more reliable benchmarking of recovery. 

A second limitation lies in the incomplete capture of defensive metrics, particularly in football and basketball. Current fantasy systems tend to emphasize offensive statistics and undervalue contributions that are not easily quantified, such as a cornerback’s coverage ability or a power forward’s screen-setting. These elements are often critical to team success but may not be reflected in fantasy point totals. To address this, future iterations of RPIP frameworks could incorporate emerging advanced metrics, such as NFL coverage grades or NBA defensive plus-minus scores, to provide a more balanced assessment of overall player impact. 

Sample size constraints also emerged as a challenge, particularly in studies examining less common injuries or smaller sports leagues. Many of the included cohorts were small, reducing statistical power and limiting the ability to detect nuanced effects across positions or injury types. Collaborative research efforts across teams, institutions, and leagues could help generate larger, more representative datasets that improve the reliability of future analyses. 

Another important limitation is the use of retrospective designs, which introduces the potential for survivorship bias. Most studies excluded non-returning athletes, potentially underestimating impacts - particularly for spine injuries like lumbar discectomy, where neural compromise may lead to higher retirement rates [19]. Prospective studies that track players from the point of injury through their return or retirement would provide a more complete and accurate picture of recovery trajectories. 

Finally, there are variations in scoring systems across fantasy platforms that, while often minor, could affect absolute performance values. Differences such as bonus points for long touchdowns, differing reception weights (e.g., full-PPR vs. half-PPR), or variations in turnover penalties may slightly shift post-injury outputs. Although these discrepancies limit direct comparability across studies, they are unlikely to materially change the overall clinical interpretation of recovery, since the core scoring structure across platforms remains consistent.

Return to pre-injury performance (RPIP): a future benchmark

Notably, while the studies in this review consistently demonstrated post-injury declines using fantasy metrics, none explicitly framed their analyses around RPIP as a defined metric. RPIP, the percentage of a player’s prior output recovered post-injury, offers a powerful, individualized, and scalable measure that goes beyond binary return-to-play endpoints. Standardizing RPIP across studies could enable more meaningful comparisons across injuries, positions, and leagues. Future research should prioritize formalizing RPIP as a core outcome, leveraging fantasy scores and WAR as accessible, longitudinal tools to benchmark recovery quality, guide rehabilitation, and inform athlete readiness. 

The implications of a standardized RPIP framework extend well beyond academic inquiry and hold the potential to reshape clinical and operational decision-making in sports. In the clinical setting, fantasy metrics could serve as objective benchmarks to monitor rehabilitation progress and help determine when an athlete is truly ready to return to competition. For players and their care teams, having access to concrete data on typical recovery patterns, for example, that NFL running backs average a 15% decline in fantasy production during their first post-ACL season, can help set realistic expectations and improve counseling. At the team level, general managers could use RPIP-based benchmarks to guide contract negotiations, allocate playing time, and plan return-to-competition timelines with greater precision. Finally, on a broader scale, identifying injury types that result in severe or prolonged performance declines, such as lumbar discectomy with reported 57-100% reductions, could inform league policy decisions, including rule changes or targeted equipment improvements. 

Looking forward, future studies could further enhance validity by integrating clinical outcome measures (e.g., ODI, VAS), advanced imaging (e.g., MRI evidence of tissue healing), and fantasy scores. Such multimodal validation would provide a stronger foundation for establishing fantasy metrics as legitimate proxies for return to pre-injury performance in spine and other injuries, while also helping bridge the gap between sport-specific analytics and clinically validated outcomes. 

To unlock these benefits, several key steps are needed to refine and expand the RPIP framework. First, applying RPIP methodologies to broader populations, such as women's professional leagues like the Women's National Basketball Association (WNBA) and National Women's Soccer League (NWSL) and collegiate athletes, would help address longstanding gaps in sports injury research. Second, integrating fantasy metrics with biomechanical and player tracking data (e.g., NFL Next Gen Stats, NBA Second Spectrum) could reveal movement-level predictors of underperformance, providing a more granular understanding of recovery. Third, developing injury- and position-specific RPIP curves would allow for individualized forecasting and return-to-play thresholds based on a player’s unique profile. Lastly, standardization efforts will be essential: establishing best practices for calculating RPIP and harmonizing fantasy scoring systems across studies will improve comparability, enhance clinical and research utility, and, where feasible, encourage the use of matched cohort designs to control for confounding factors and strengthen causal inference. 

## Conclusions

This narrative review underscores the value of fantasy sports metrics and WAR as meaningful tools for assessing post-injury performance across professional sports. In a landscape where RTP has traditionally served as the default recovery milestone, these composite metrics offer a more nuanced, performance-centered alternative. Across the included studies, spanning sports, injuries, and methodologies, fantasy scores and WAR consistently reflected post-injury declines, reinforcing their validity as scalable, data-rich indicators of recovery. Although no study explicitly framed their analysis around RPIP, the structure and outcomes of these investigations provide a strong foundation for formalizing RPIP as a future benchmark. By aggregating diverse elements of player productivity into a single score, fantasy metrics offer a standardized and comparable method to quantify RPIP across positions, injury types, and leagues - something no single statistic or subjective evaluation can achieve alone. 

While limitations remain, including variability in sample sizes, scoring systems, and post-injury performance windows, the consistency of findings presents an important opportunity. Standardizing RPIP, encouraging matched cohort designs, and integrating this metric into future research could help clinicians, teams, and athletes make more informed, performance-oriented recovery decisions. In an era defined by increasingly granular data, fantasy scores and WAR stand out as underutilized but highly promising tools. They provide a potentially reliable method to evaluate what matters most: not merely whether athletes return, but the extent to which they are able to reclaim their prior performance once they do.
